# Supplementary material for: Genomic dissection of endemic carbapenem resistance reveals metallo-beta-lactamase dissemination through clonal, plasmid and integron transfer
Source: Nat Commun. 2023 Aug 8;14:4764. doi: 10.1038/s41467-023-39915-2 (PMC10409761; doi:10.1038/s41467-023-39915-2)
Supplement: Supplementary file 1 — Supplementary Information [file 41467_2023_39915_MOESM1_ESM.pdf]

Supp. Table 1 – *bla*$_{IMP-4}$ plasmid types with corresponding MOB-typer clusters and plasmid taxonomic units

Plasmid type	MOB-typer primary cluster	MOB-typer secondary cluster	Plasmid taxonomic unit (PTU)
IncC	AA860	AJ266	PTU-C
IncFIA/IncFIB/IncP	AD444	AN003	PTU could not be assigned
IncFIB	AA020	AH567	PTU could not be assigned
IncHI2A type 1	AA739	AJ055	PTU-HI2
IncHI2A type 2	AA739	AJ058	PTU-HI2
IncL/M	AA002	AH532	PTU-L/M
Untypeable	AC935	AM306	PTU could not be assigned

Abbreviations: PTU – plasmid taxonomic unit.

Supp. Table 2 – Phylogenetic distance analysis of key *bla*$_{IMP-4}$ bacterial host strains from the Alfred Hospital

Bacterial strain	Mean pairwise SNV distance	Median pairwise SNV distance	Interquartile range
Enterobacter hormaechei ST114	39.3	35	27-46
Enterobacter hormaechei ST190	13.6	3	2-4
Enterobacter hormaechei ST93	9.6	9	2-14
Klebsiella oxytoca ST278	2	2	1-3
Klebsiella pneumoniae ST4379	31.2	31.5	14.25-48.75
Pseudomonas aeruginosa ST111	1.6	1	0-2
Serratia marcescens lineage 1	7.8	8	4-11
Serratia marcescens lineage 2	1	1	1-1

Abbreviations: SNV – single nucleotide variant, ST – sequence type.

Supp. Table 3 – Distance metrics for key *bla*$_{IMP-4}$ plasmid types

Plasmid type<br>MOB-typer cluster		IncC<br>(AA860 AJ266)	IncFIA / IncFIB / IncP<br>(AD444 AN003)	IncHI2A type 1<br>(AA739 AJ055)	IncHI2A type 2<br>(AA739 AJ058)	IncL/M<br>(AA002 AH532)	Untypeable<br>(AC935 AM306)
**Mash distances**	Median distance	2.4444×10^{-3}	99.33×10^{-55}	99.49×10^{-4}	33.07×10^{-4}	5.18×10^{-4}	77.91×10^{-3}
	Mean distance	4.0303×10^{-3}	2562.56×10^{-22}	1.49×10^{-3}	44.37×10^{-4}	7.3535×10^{-4}	66.17×10^{-22}
	Interquartile range	6.8989×10^{-4}—5.66×10^{-3}	5.57×10^{-5}–9–9.58×10^{-22}	2.0303×10^{-4}—1.4646×10^{-3}	11.95×10^{-4}–6–6.57×10^{-4}	11.17×10^{-5}—1.09×10^{-3}	66.00×10^{-3}–00.143
**SNV analysis**	Total core alignment SNV sites	11	2	25	5	5	1122
	Median pairwise SNV distance	0	0	0	0	0	1013.5
	Mean pairwise SNV distance	0.39	0.25	1.88	0.33	0.69	718.83
	Interquartile range	0 – 1	0 – 0	0 – 4	0 – 1	0 – 1	373 – 1034.5
**Pairwise average nucleotide identity**	Median nucleotide identity	99.83%	99.9595%	99.95%	99.9797%	99.9595%	99.3434%
	Mean nucleotide identity	99.7777%	982398.23%	99.9393%	99.97%	99.9494%	99.4646%
	Interquartile range	99.7373% – 99.8989%	95.48% – 99.98%	99.8888% – 99.9797%	99.96% – 99.9898%	99.93% – 100%	99.189918% – 99.80%

Abbreviations: SNV – single nucleotide variant

Supp. Table 4 – Characteristics of *bla*$_{IMP-4}$ mosaic plasmids

Isolate	Bacterial host strain	Flanking region cluster	Integron SNV profile	*bla*$_{IMP-4}$ genetic setting	Plasmid replicon types present	MOB-typer primary cluster	MOB-typer secondary cluster
CPO136	*Klebsiella pneumoniae* ST340	14	N/A	IncC	IncC, IncFIB	AA860	AJ266
CPO150	*Klebsiella pneumoniae* ST340	A	GCATCGACGCCT	IncC	IncC, IncFIB	AA860	AJ266
CPO161	*Klebsiella pneumoniae* ST4379	11	N/A	IncC	IncC, ColRNA (rep cluster 1987), IncP	AA860	AJ266
CPO328	*Klebsiella variicola* ST1582	E	TGGTCGGCGCCT	IncC	IncC, IncN	AA860	AJ266
CPO422	*Serratia marcescens* lin. 1	A	TGGTCGACGCCT	IncC	IncC, ColRNA (rep cluster 1987)	AA860	AJ266
CPO475	*Serratia marcescens* lin. 1	B	TGGTCGACGCCT	IncC	IncC, rep cluster 2335	AA860	AJ266
CPO504	*Klebsiella pneumoniae* ST1552 (1LV)	A	TGGTCGACGCCT	IncC	IncC, IncFIA, IncFIB, IncFIC	AA861	AJ282
CPO044	*Enterobacter hormaechei* ST190	F	GGGTCGACGTCT	IncHI2	IncHI2A, IncR, rep cluster 1088	AA739	AJ055
CPO062	*Enterobacter hormaechei* ST190	7	GGGTCGACGTCT	IncHI2	IncHI2A, IncR, rep cluster 1088	AA739	AJ055
CPO235	*Escherichia coli* ST155	F	GGGATGACGTCT	IncHI2	IncHI2A, IncX1, rep cluster 1088	AA739	AJ058

Abbreviations: lin. – lineage; LV – locus variant; ST – Sequence type; N/A – not applicable due to insertions or deletions in integron structure

Supp. Table 5 – Integron single nucleotide variant profiles in *bla*$_{\text{IMP-4}}$ plasmids

Integron SNV Profile	Total plasmids	Genetic setting associated with integron SNV profile
TGGTCGACGCCT	75	Chromosome, IncC, IncFIA/IncFIB/IncP, IncFIB
GGGTCGACGTCT	38	Chromosome, IncHI2A type 1, IncHI2A type 2
GGGTCGACGCCT	25	Chromosome, IncC, IncHI2A type 1, IncL/M
GGGTCGACACCT	4	IncHI2A type 1
GGGATGACGTCT	2	IncHI2A type 1, IncHI2A type 2
GCATCGACGCCT	1	IncC
TGGTCGGCGCCT	1	IncC
TGGTTGACGCCT	1	IncC
TGGTCGACGCTC	1	IncFIA/IncFIB/IncP
GGGTCGATGTCT	1	IncHI2A type 1
GGGATTACGTCT	1	IncHI2A type 2
GGGTCTACGTCT	1	IncHI2A type 2

Abbreviations: SNV – single nucleotide variant.

Supp. Fig. 1 – Epidemiological curve of *bla*$_{IMP-4}$ genetic settings

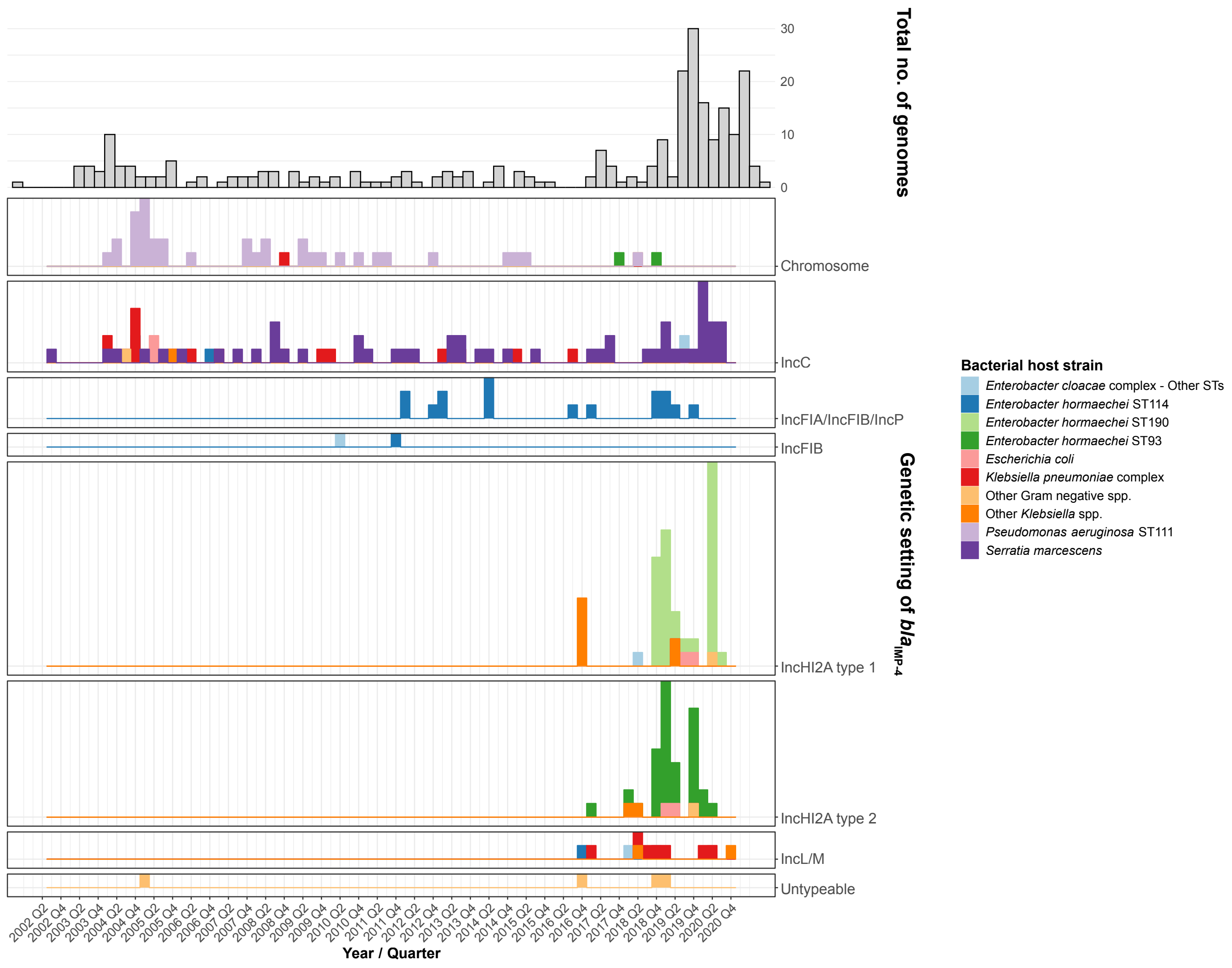

Top panel indicates overall number of *bla*$_{IMP-4}$ genomes during each year/quarter. Bottom panels show *bla*$_{IMP-4}$ genomes per genetic setting (shown as separate panels) and bacterial host strain (shown as different colours).

Abbreviations: No. – number; ST – sequence type.

Supp. Fig. 2 – Phylogenetic analysis of key *bla*$_{IMP-4}$ bacterial host strains

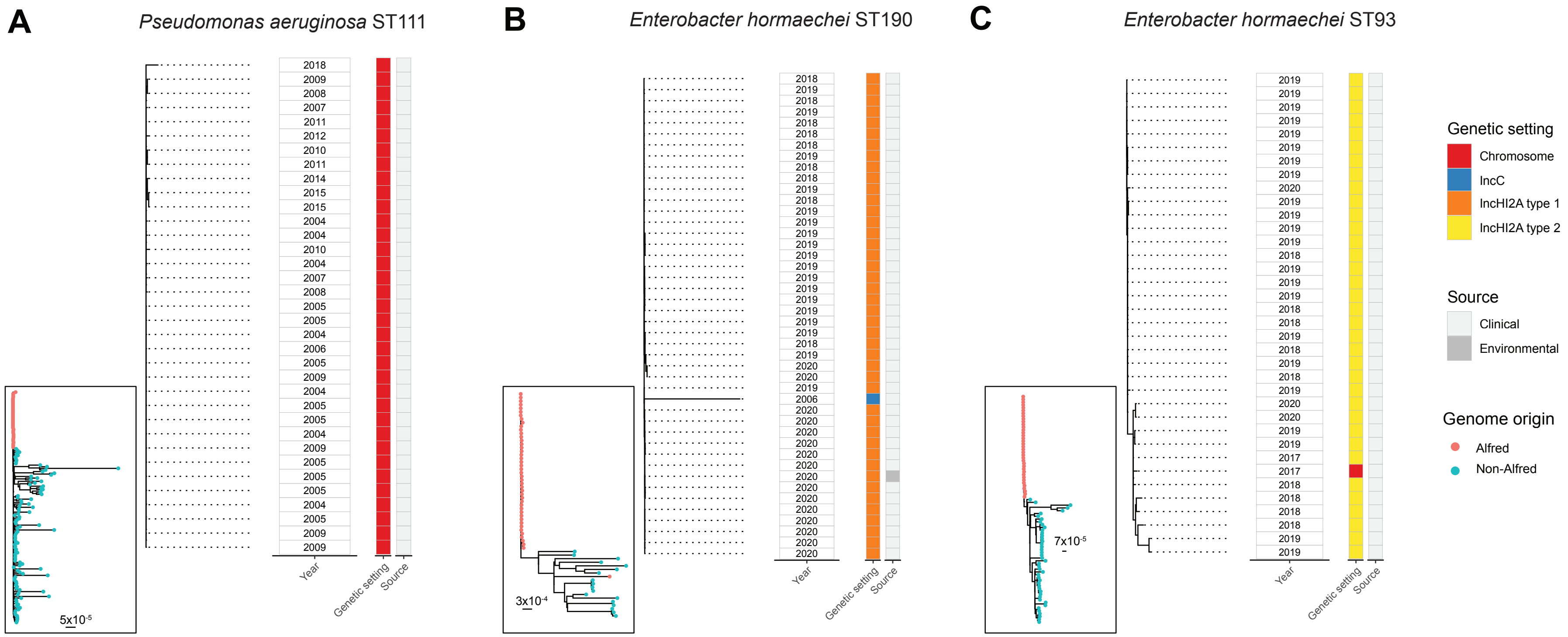

The inset panels locate genomes from the Alfred Hospital in phylogenies of global publicly available genomes of the same sequence type, with the outer panel showing Alfred Hospital genomes only. Genomes for each of these three strains were clonally related, with median pairwise single nucleotide variant distances <20.
Supp. Fig. 2a: Phylogenies for *Pseudomonas aeruginosa* ST111.
Supp. Fig. 2b: Phylogenies for *Enterobacter hormaechei* ST190.
Supp. Fig. 2c: Phylogenies for *Enterobacter hormaechei* ST93.

Abbreviations: ST – Sequence type.

Supp. Fig. 3 – *bla*$_{IMP-4}$ flanking clusters and integron single nucleotide variant profiles by plasmid type

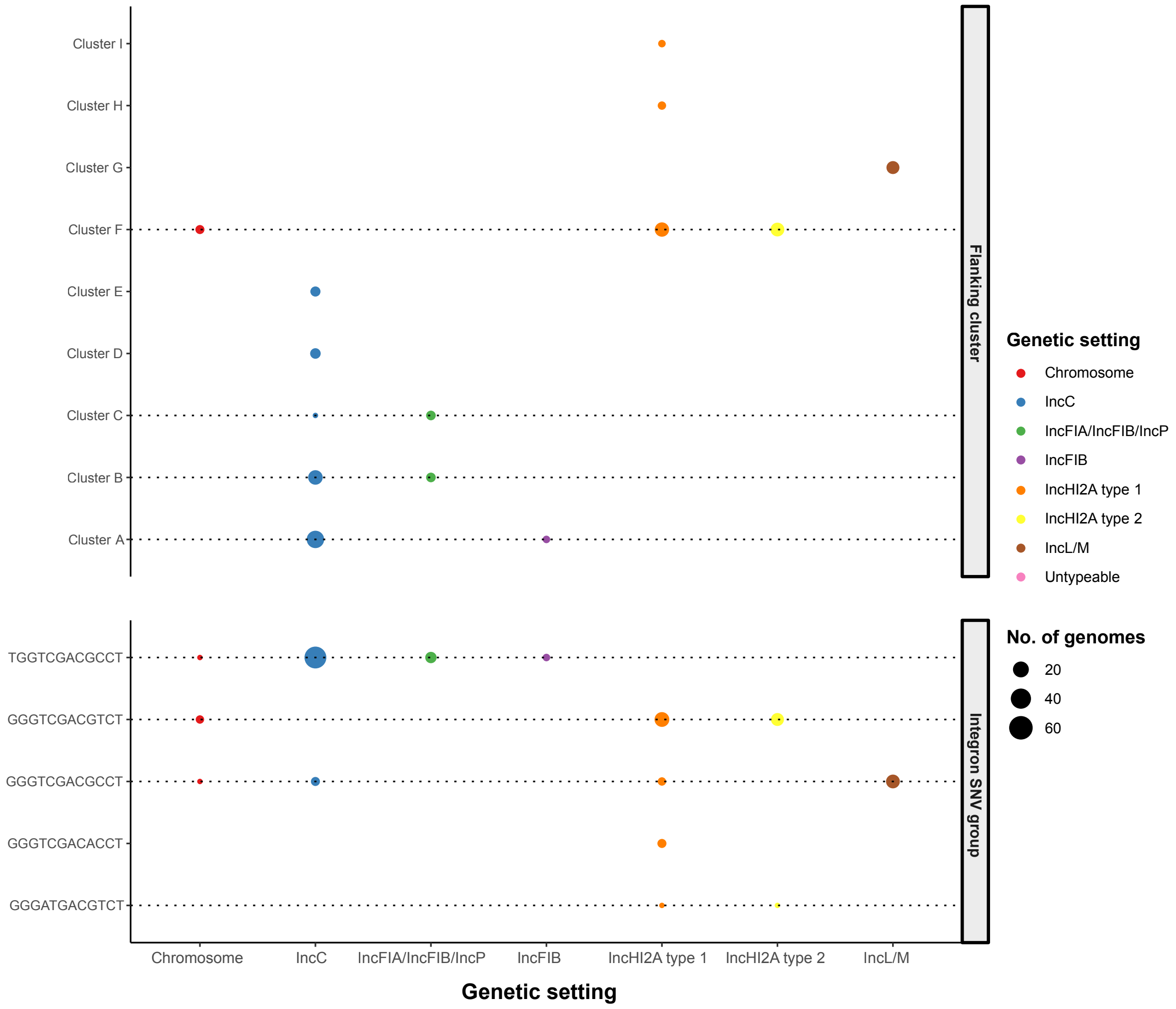

Top panel shows flanking clusters and bottom panel shows integron single nucleotide variant (SNV) profiles. Dotted lines represent flanking clusters or integron SNV profiles that were shared across different genetic settings, as represented by different point colours. Point size indicates the number of genomes.

Abbreviations: No. – number; SNV – single nucleotide variant.
