# Supplementary material for: Genomic dissection of endemic carbapenem resistance reveals metallo-beta-lactamase dissemination through clonal, plasmid and integron transfer
Source: Nat Commun. 2023 Aug 8;14:4764. doi: 10.1038/s41467-023-39915-2 (PMC10409761; doi:10.1038/s41467-023-39915-2)
Supplement: Supplementary file 3 — Description of Additional Supplementary Files [file 41467_2023_39915_MOESM3_ESM.pdf]

Description of Additional Supplementary Files

Supplementary Dataset 1: ‘Abbreviations: LV - locus variant; NA - Not applicable; No. - number; SNV - single nucleotide variant; SRA - Sequence Read Archive.’
