# Supplementary material for: Genomic dissection of endemic carbapenem resistance reveals metallo-beta-lactamase dissemination through clonal, plasmid and integron transfer
Source: Nat Commun. 2023 Aug 8;14:4764. doi: 10.1038/s41467-023-39915-2 (PMC10409761; doi:10.1038/s41467-023-39915-2)
Supplement: Supplementary file 5 — Reporting Summary [file 41467_2023_39915_MOESM5_ESM.pdf]

nature portfolio

Corresponding author(s): Anton Y. Peleg

Last updated by author(s): 18/06/2023

Reporting Summary

Nature Portfolio wishes to improve the reproducibility of the work that we publish. This form provides structure for consistency and transparency in reporting. For further information on Nature Portfolio policies, see our Editorial Policies and the Editorial Policy Checklist.

Statistics

For all statistical analyses, confirm that the following items are present in the figure legend, table legend, main text, or Methods section.

n/a	Confirmed	
☐	☒	The exact sample size (*n*) for each experimental group/condition, given as a discrete number and unit of measurement
☒	☐	A statement on whether measurements were taken from distinct samples or whether the same sample was measured repeatedly
☒	☐	The statistical test(s) used AND whether they are one- or two-sided<br>*Only common tests should be described solely by name; describe more complex techniques in the Methods section.*
☐	☒	A description of all covariates tested
☐	☒	A description of any assumptions or corrections, such as tests of normality and adjustment for multiple comparisons
☐	☒	A full description of the statistical parameters including central tendency (e.g. means) or other basic estimates (e.g. regression coefficient) AND variation (e.g. standard deviation) or associated estimates of uncertainty (e.g. confidence intervals)
☒	☐	For null hypothesis testing, the test statistic (e.g. *F*, *t*, *r*) with confidence intervals, effect sizes, degrees of freedom and *P* value noted<br>*Give P values as exact values whenever suitable.*
☒	☐	For Bayesian analysis, information on the choice of priors and Markov chain Monte Carlo settings
☒	☐	For hierarchical and complex designs, identification of the appropriate level for tests and full reporting of outcomes
☒	☐	Estimates of effect sizes (e.g. Cohen's *d*, Pearson's *r*), indicating how they were calculated

Our web collection on statistics for biologists contains articles on many of the points above.

Software and code

Policy information about availability of computer code

Data collection	Guppy v.4.0.14 was used for base-calling of long-read sequencing.
Data analysis	The code generated during this study has been deposited in the following GitHub repository: https://github.com/nenadmacesic/imp4_ncomms (DOI:10.5281/zenodo.8051453)<br><br>The following software tools were used during data analysis:<br>R v4.1.1, Shovill v1.0.4, Filtlong v.0.2.0, Unicycler v.0.4.08, Flye v2.9.2, Medaka v1.8.0, Polypolish v0.5.0, Polca v3.4.1, Quast v5.2.0, GTDB-Tk v1.0.2, Prokka v1.14.6, Abricate v.1.0.0, mlst v.2.19.0, SRST2 v0.2.0, Assembly Dereplicator v0.1.0, Mashtree v1.2.0, PHASTER (web server - https://phaster.ca/), Island Viewer 4 (web server - https://www.pathogenomics.sfu.ca/islandviewer/), Snippy v.4.6.0, Gubbins v3.3, IQtree v.2.0.3, Adobe Illustrator v2020.24.3, MOB-typer v1.4.9, COPLA (web server - https://castillo.dicom.unican.es/copla/), ggtree v3.0.4, fastANI v1.3, Mauve v2.4.0.r4736, Easyfig v2.2.2, Flanker v0.1.5, Geneious v10.2.6, MUSCLE v3.8.1551, SNP-sites v2.5.1, REDDog pipeline V1.beta10.3, ggraph v2.0.5, iGraph v1.2.11.

For manuscripts utilizing custom algorithms or software that are central to the research but not yet described in published literature, software must be made available to editors and reviewers. We strongly encourage code deposition in a community repository (e.g. GitHub). See the Nature Portfolio guidelines for submitting code & software for further information.

Data

Policy information about availability of data

All manuscripts must include a data availability statement. This statement should provide the following information, where applicable:

- Accession codes, unique identifiers, or web links for publicly available datasets
- A description of any restrictions on data availability
- For clinical datasets or third party data, please ensure that the statement adheres to our policy

Illumina/Nanopore read data were deposited in the NCBI SRA under project accession PRJNA924056. Completed genome assemblies were deposited in GenBank; accessions are listed in Supp. Table 1 and are listed below:

Isolate GenBank accession no.
CPO106 GCA_030186425.1
CPO118 GCA_016774035.1
CPO058 GCA_016774245.1
CPO020 GCA_030187455.1
CPO100 GCA_030186885.1
CPO532 GCA_030183065.1
CPO495 GCA_030183425.1
CPO104 GCA_030186815.1
CPO573 GCA_030182705.1
CPO459 GCA_030183855.1
CPO460 GCA_030183815.1
CPO463 GCA_030183795.1
CPO158 GCA_030186035.1
CPO139 GCA_030186285.1
CPO493 GCA_030183495.1
CPO494 GCA_030183575.1
CPO465 GCA_030183755.1
CPO469 GCA_030183745.1
CPO471 GCA_030183695.1
CPO475 GCA_030183675.1
CPO478 GCA_030183635.1
CPO482 GCA_030183605.1
CPO486 GCA_030183595.1
CPO522 GCA_030183175.1
CPO487 GCA_030183545.1
CPO492 GCA_030183515.1
CPO513 GCA_030183275.1
CPO491 GCA_030183535.1
CPO500 GCA_030183445.1
CPO520 GCA_030183245.1
CPO521 GCA_030183135.1
CPO534 GCA_030183005.1
CPO529 GCA_030183035.1
CPO538 GCA_030182995.1
CPO537 GCA_030183075.1
CPO540 GCA_030182975.1
CPO546 GCA_030182885.1
CPO547 GCA_030182905.1
CPO543 GCA_030182875.1
CPO542 GCA_030182925.1
CPO545 GCA_030182955.1
CPO552 GCA_030182795.1
CPO163 GCA_030186095.1
CPO554 GCA_030182785.1
CPO555 GCA_030182775.1
CPO180 GCA_030185815.1
CPO563 GCA_030182815.1
CPO111 GCA_030186385.1
CPO093 GCA_030186475.1
CPO109 GCA_030169065.1
CPO229 GCA_030185435.1
CPO242 GCA_030185275.1
CPO103 GCA_030186875.1
CPO140 GCA_030186235.1
CPO067 GCA_030187085.1
CPO105 GCA_030186495.1
CPO107 GCA_030186435.1
CPO185 GCA_030185675.1
CPO186 GCA_030185655.1
CPO082 GCA_016773175.1

CPO251 GCA_030185155.1
CPO267 GCA_030184965.1
CPO286 GCA_030184835.1
CPO187 GCA_030185635.1
CPO570 GCA_030182805.1
CPO098 GCA_030186935.1
CPO181 GCA_030185775.1
CPO156 GCA_030186055.1
CPO009 GCA_016772915.1
CPO253 GCA_030185195.1
CPO053 GCA_030187275.1
CPO088 GCA_016774315.1
CPO403 GCA_030183985.1
CPO075 GCA_030186995.1
CPO074 GCA_030187035.1
CPO496 GCA_030183455.1
CPO141 GCA_030186255.1
CPO072 GCA_030187135.1
CPO504 GCA_030183345.1
CPO323 GCA_030184735.1
CPO324 GCA_030184595.1
CPO010 GCA_030187555.1
CPO190 GCA_030185685.1
CPO214 GCA_030185535.1
CPO055 GCA_016774235.1
CPO011 GCA_030187575.1
CPO189 GCA_030185735.1
CPO174 GCA_030185915.1
CPO142 GCA_030186275.1
CPO145 GCA_016773055.1
CPO182 GCA_030185795.1
CPO183 GCA_030185755.1
CPO173 GCA_030185925.1
CPO064 GCA_030187175.1
CPO069 GCA_030187115.1
CPO176 GCA_030185865.1
CPO092 GCA_016773015.1
CPO143 GCA_030186205.1
CPO160 GCA_030186015.1
CPO120 GCA_030186415.1
CPO132 GCA_030186345.1
CPO117 GCA_030186825.1
CPO157 GCA_030186105.1
CPO151 GCA_030186135.1
CPO153 GCA_030186075.1
CPO119 GCA_016773035.1
CPO129 GCA_016774135.1
CPO136 GCA_030186855.1
CPO137 GCA_030186295.1
CPO150 GCA_030186155.1
CPO248 GCA_030185255.1
CPO402 GCA_030184015.1
CPO147 GCA_030186195.1
CPO287 GCA_030184945.1
CPO128 GCA_030186795.1
CPO243 GCA_030185295.1
CPO249 GCA_030185235.1
CPO123 GCA_030186375.1
CPO116 GCA_016772995.1
CPO071 GCA_030187125.1
CPO094 GCA_016774155.1
CPO400 GCA_030184055.1
CPO399 GCA_016774175.1
CPO397 GCA_030184075.1
CPO401 GCA_030184035.1
CPO386 GCA_030184115.1
CPO389 GCA_030184135.1
CPO056 GCA_030187205.1
CPO008 GCA_030187595.1
CPO216 GCA_030185505.1
CPO280 GCA_030184895.1
CPO052 GCA_016773135.1
CPO191 GCA_030185595.1
CPO023 GCA_030187525.1
CPO203 GCA_030185555.1
CPO210 GCA_030185575.1

CPO076 GCA_030187025.1
CPO078 GCA_030186985.1
CPO079 GCA_030186975.1
CPO044 GCA_016774285.1
CPO004 GCA_030187725.1
CPO073 GCA_030187075.1
CPO013 GCA_030187515.1
CPO221 GCA_030185455.1
CPO224 GCA_030185465.1
CPO263 GCA_030185015.1
CPO051 GCA_016774195.1
CPO045 GCA_030187295.1
CPO046 GCA_030187255.1
CPO016 GCA_030187475.1
CPO002 GCA_030187635.1
CPO259 GCA_030185075.1
CPO298 GCA_030184785.1
CPO338 GCA_030184495.1
CPO353 GCA_030184475.1
CPO054 GCA_030187235.1
CPO068 GCA_016774265.1
CPO029 GCA_016774045.1
CPO024 GCA_030187395.1
CPO066 GCA_016773095.1
CPO049 GCA_016773155.1
CPO050 GCA_030187195.1
CPO014 GCA_030187485.1
CPO328 GCA_030184575.1
CPO033 GCA_030187355.1
CPO021 GCA_030187435.1
CPO042 GCA_016773195.1
CPO254 GCA_030185175.1
CPO369 GCA_030184275.1
CPO241 GCA_030185395.1
CPO250 GCA_030185205.1
CPO256 GCA_030185115.1
CPO234 GCA_030185415.1
CPO235 GCA_030185365.1
CPO239 GCA_030185345.1
CPO312 GCA_030184635.1
CPO264 GCA_030184995.1
CPO285 GCA_030184875.1
CPO255 GCA_030185135.1
CPO260 GCA_030185045.1
CPO261 GCA_030185065.1
CPO262 GCA_030185035.1
CPO272 GCA_030184935.1
CPO278 GCA_030184915.1
CPO291 GCA_030184815.1
CPO292 GCA_030184775.1
CPO344 GCA_030184515.1
CPO335 GCA_030184535.1
CPO303 GCA_030184655.1
CPO301 GCA_030184755.1
CPO302 GCA_030184715.1
CPO320 GCA_030184615.1
CPO321 GCA_030184665.1
CPO337 GCA_030184555.1
CPO355 GCA_030184315.1
CPO356 GCA_030184435.1
CPO305 GCA_030184675.1
CPO360 GCA_030184415.1
CPO362 GCA_030184395.1
CPO347 GCA_030184445.1
CPO365 GCA_030184335.1
CPO367 GCA_030184375.1
CPO405 GCA_030183975.1
CPO422 GCA_030183895.1
CPO019 GCA_016772875.1
CPO134 GCA_030186335.1
CPO086 GCA_030186915.1
CPO526 GCA_030183145.1
CPO505 GCA_030183325.1
CPO040 GCA_016773995.1
CPO089 GCA_016773065.1
CPO240 GCA_030185335.1

CPO458 GCA_030183835.1
CPO081 GCA_030186955.1
CPO121 GCA_016774075.1
CPO003 GCA_030187655.1
CPO220 GCA_030185495.1
CPO244 GCA_030185315.1
CPO101 GCA_030186595.1
CPO017 GCA_016772905.1
CPO006 GCA_030187615.1
CPO062 GCA_016774215.1
CPO175 GCA_030185895.1
CPO112 GCA_030248925.1
CPO506 GCA_030183355.1
CPO161 GCA_016774095.1
CPO368 GCA_030184295.1
CPO370 GCA_030184255.1
CPO477 GCA_030183655.1
CPO566 GCA_030182735.1
CPO022 GCA_030187405.1
CPO361 GCA_030184345.1
CPO385 GCA_030184155.1
CPO102 GCA_030186505.1
CPO167 GCA_030185975.1
CPO149 GCA_030186165.1
CPO519 GCA_030183195.1
CPO184 GCA_030185715.1
CPO170 GCA_030185965.1
CPO060 GCA_016773115.1
CPO371 GCA_030184215.1
CPO429 GCA_030183875.1
CPO474 GCA_030183715.1
CPO470 GCA_030183735.1
CPO497 GCA_030183415.1
CPO503 GCA_030183335.1
CPO509 GCA_030183315.1
CPO510 GCA_030183295.1
CPO512 GCA_030183235.1
CPO514 GCA_030183215.1
CPO523 GCA_030183115.1
CPO528 GCA_030183055.1
CPO188 GCA_030185615.1
CPO178 GCA_016772975.1
CPO179 GCA_030185855.1
CPO177 GCA_030185835.1
CPO165 GCA_030185955.1
CPO166 GCA_016772955.1
CPO171 GCA_016774115.1
CPO037 GCA_030187375.1
CPO038 GCA_030187335.1
CPO039 GCA_030187305.1
CPO284 GCA_030184855.1
CPO373 GCA_030184225.1
CPO377 GCA_030184195.1
CPO382 GCA_030184165.1
CPO395 GCA_030184095.1
CPO410 GCA_030183955.1
CPO411 GCA_030183935.1
CPO417 GCA_030183915.1

Research involving human participants, their data, or biological material

Policy information about studies with human participants or human data. See also policy information about sex, gender (identity/presentation), and sexual orientation and race, ethnicity and racism.

Reporting on sex and gender	Sex and gender data were not collected as not relevant to the study.
Reporting on race, ethnicity, or other socially relevant groupings	Race/ethnicity/socially relevant grouping data were not collected as not relevant to the study.
Population characteristics	No covariant-relevant population characteristics were relevant or taken into account in this study.
Recruitment	We included all patients whose isolates had been sequenced according to the approach outlined in 'Sample Size' below.

Ethics oversight	The study was approved by the Alfred Hospital Ethics Committee (Project No: 44/20)

Note that full information on the approval of the study protocol must also be provided in the manuscript.

Field-specific reporting

Please select the one below that is the best fit for your research. If you are not sure, read the appropriate sections before making your selection.

☒ Life sciences ☐ Behavioural & social sciences ☐ Ecological, evolutionary & environmental sciences

For a reference copy of the document with all sections, see nature.com/documents/nr-reporting-summary-flat.pdf

Life sciences study design

All studies must disclose on these points even when the disclosure is negative.

Sample size	We selected 277 blaIMP-4 isolates for whole genome sequencing (WGS) based on bacterial strain (species/MLST combination) and year of isolation. No sample size calculation was performed. Sample sizes were sufficient because species with <30 isolates had all isolates sequenced and characterised. For species with >30 isolates, we performed WGS on selected isolates based on collection date to ensure that we had sequencing data available for all study periods. This decision was made because their were periods of significant clonal spread, with large numbers of highly similar genomes.
Data exclusions	No data were excluded.
Replication	No experiments requiring replication were performed as all experiments were bioinformatic in nature.
Randomization	Randomization was not relevant since this was an observational study and we included samples of all the patients according to the approach outlined in 'Sample Size' below.
Blinding	Blinding was not needed as this was an observational study.

Reporting for specific materials, systems and methods

We require information from authors about some types of materials, experimental systems and methods used in many studies. Here, indicate whether each material, system or method listed is relevant to your study. If you are not sure if a list item applies to your research, read the appropriate section before selecting a response.

Materials & experimental systems

n/a	Involved in the study	
☒	☐	Antibodies
☒	☐	Eukaryotic cell lines
☒	☐	Palaeontology and archaeology
☒	☐	Animals and other organisms
☒	☐	Clinical data
☒	☐	Dual use research of concern
☒	☐	Plants

Methods

n/a	Involved in the study	
☒	☐	ChIP-seq
☒	☐	Flow cytometry
☒	☐	MRI-based neuroimaging
